# A new genus of Languriinae from Mexico (Coleoptera, Erotylidae), with comments on the potential mimic phenomenon of some languriines

**DOI:** 10.3897/zookeys.935.49957

**Published:** 2020-05-21

**Authors:** Zheng-Zhong Huang, Xing-Ke Yang, Si-Qin Ge

**Affiliations:** 1 Insititute of Zoology, Chinese Academy of Sciences, 1 Beichen West Road, Chaoyang District, Beijing, 100101, China Insititute of Zoology, Chinese Academy of Sciences Beijing China

**Keywords:** Cucujoidea, Neotropical Region, new combination, *
Tomolanguria
*

## Abstract

A new genus of Languriinae, *Tomolanguria* Huang, **gen. nov.** is erected for a single species, *Languria aculeata* Gorham, 1887 from Mexico. It is similar to the Neotropical genus *Languria* Latreille, 1802. The differential diagnosis of this new genus is based on the structural features of the elytral apices and slight impressions present on each elytron. *Languria aculeata* is designated as the type species of the new genus. This species is redescribed and illustrated (all the three specimens examined are shown in the dorsal, ventral, and lateral views). Finally, a mimic relationship between this new genus and genus *Paederus* Fabricius, 1775 (Staphylinidae) is discussed.

## Introduction

*Languria aculeata* was first described by Gorham (1887), based on three specimens in the Sallé collection. Martins and Perreira (1965) accomplished a revision of the Neotropical Languriinae, but they did not examine the specimens of *L. aculeata* and just cited Gorham’s original description in their work. In 2017, we had a chance to examine specimens of Languriinae, including the syntypes of *L. aculeata*, in the Natural History Museum, London. The elytral structure of *L. aculeata* clearly separates this species from all known Neotropical genera of Languriinae. Hence, we propose a new genus and a new combination for its type species.

## Materials and methods

The type series of *Tomolanguria* gen. nov., *Anadastus ornatus* Arrow, 1925, *A. pulchellus* Arrow, 1925, *A. jucundus* (Gorham, 1903), *Clerolanguria tricolor* (Fabricius, 1787), and *Stenolanguria tricolor* Fowler, 1885 are deposited in the Natural History Museum, London, United Kingdom (**BMNH**). The holotypes of *Paederolanguria holdhausi* Mader, 1939 and *P. klapperichi* Mader, 1955 are deposited in the Naturhistorisches Museum, Basel, Switzerland (**NHMB**). The holotype of *Paederolanguria alternata* (Zia, 1959) and non-type specimen of *P. holdhausi* are deposited in the Institute of Zoology of the Chinese Academy of Sciences, Beijing, China (**IZCAS**). The holotype of *Stenolanguria robusta* Villiers, 1958 is deposited in the Muséum national d’Histoire naturelle, Paris, France (**MNHN)**. Label data are given with separate lines on labels indicated by / and separate labels by //. Other comments and remarks are in square brackets [].

All photographs were taken with a Canon 5D Mark III digital camera equipped with a Canon MP-E 65 mm lens. The images were stacked with Helicon Focus 6.7.1 and modified in Adobe Photoshop CS6 to correct for contrast, brightness, and imperfections.

Body length was measured from the apices of mandibles to the apices of elytra.

## Taxonomy

### Family Erotylidae Latreille, 1802

**Subfamily Languriinae Hope, 1840**

**Tribe Languriini Hope, 1840**

#### Genus Tomolanguria

Taxon classificationAnimaliaColeopteraErotylidae

Huang gen. nov.

7575E56D-4007-5629-818C-230D8C844D59

http://zoobank.org/9EBD53DE-64BC-4284-8E6E-81BDB8098B34

##### Type species.

*Languria aculeata* Gorham, 1887.

##### Diagnosis.

The new genus is a member of tribe Languriini, based on the presence of a frontoclypeal suture; the antennal club composed of more than three antennomeres and relatively oval in cross-section. The only species of *Tomolanguria* can be separated from other languriine genera by the following combination of characters: body length 8.5–9.5 mm, body slender without unified metallic luster, at least prothorax without any metallic luster; antennal club not very dilated; eyes moderate in size and with fine facets; mandibles similar to each other; pronotum finely punctured, without basal foveae; lateral sides of pronotum rounded and prothorax more or less subcylindric, not flattened; elytral epipleura distinct, each elytron with transverse and very weak depressions, apices of elytra being produced and strongly tapering, rounded and with several small denticles.

##### Comparision.

The elytral structure, which often shows the differences among genera of Languriinae, is a relatively reliable morphological character. *Tomolanguria* is closely related to genus *Languria* Latreille, 1802, sharing with the latter a similar external appearance. From *Languria*, it can be distinguished by having the antennal club not very dilated; lateral sides of pronotum rounded and prothorax more or less subcylindric; each elytron with transverse and weak depressions, and apices of elytra being more or less produced and strongly tapering, and also with several small denticles. However, the elytral apex of *Languria* is simply rounded and somewhat tapering, and neither produced nor with denticles. *Tomolanguria* also resembles the Neotropical genera *Acropteroxys* Gorham, 1887 and *Langurites* Motschulsky, 1860. *Tomolanguria* differs from *Acropteroxys* in having the pronotum finely punctured, with lateral side rounded, and apices of the elytra produced and bearing small denticles. *Tomolanguria* differs from *Langurites* in having the lateral side of pronotum rounded and the apices of the elytra with neither a sharp sutural angle nor an outer angle.

On the other hand, a similar structure of the elytra is demonstrated in the Oriental genus *Paederolanguria* Mader, 1939 and African genus *Stenolanguria* Fowler, 1885 (Fig. 3G, H). The new genus can be easily separated from the former by the following characters: 1) elytral epiplerura broader and more distinct; 2) apex of prosternal process bent sharply downward (Fig. 2G, H); 3) mesoventrite without punctation and nearly smooth (Fig. 2B, C); 4) apex of elytra produced and with several small denticles (Fig. 2I, J); 5) abdomen without postmetacoxal lines. (Fig. 2D, E). *Tomolanguria* can be easily separated from *Stenolanguria* in having the head with fine punctures and the apex of elytra rounded; in *Stenolanguria*, the sutural angle is not produced and the outer angle of the elytra is produced and sharp, without denticles.

##### Etymology.

The name is derived from the Greek word *Τομός* (Latin transliteration as *tomós*, meaning “sharp”, referring to the apex of the elytra) and the generic name *Languria* (as in *Paederolanguria*, *Megalanguria*, *Caenolanguria*, etc.). Gender feminine.

##### Distribution.

Mexico, San Andrés Tuxtla.

##### Included taxa.

Only one species.

#### Tomolanguria aculeata

Taxon classificationAnimaliaColeopteraErotylidae

(Gorham, 1887) comb. nov.

C73298A4-3914-514B-A553-D98ABABE43D3

Figure 1A–L

Languria aculeata : Gorham 1887: 11, tab. I, fig 17; Fowler 1908: 29; Schenkling 1928: 16; Blackwelder 1945: 426; Martins and Perreira 1965: 154.

##### Type locality.

Mexico, San Andrés Tuxtla.

##### Redescription.

Body length 8.5–9.5 mm. Body narrow and elongate, moderately convex. Integument finely and sparsely punctured. Head pitch black. Antenna brown except for last five antennomeres yellowish. Prothorax orange. Basal third of elytra coppery green or metallic blue, remainder deep brown or black, without metallic luster.

**Figure 1.**
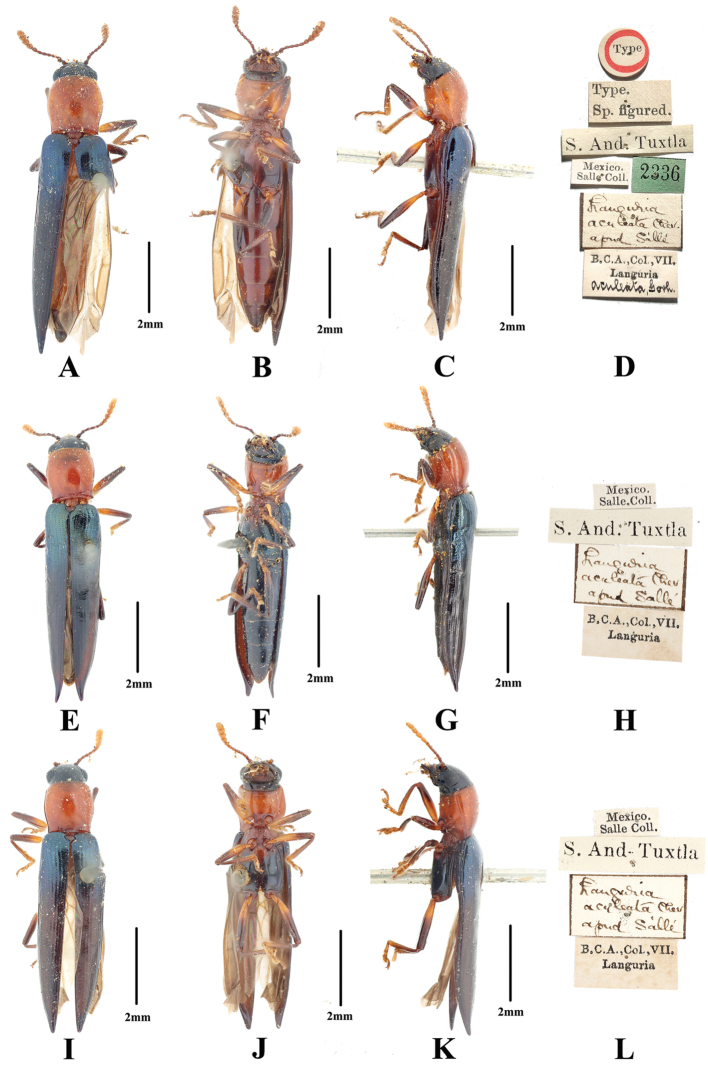
*Tomolanguria aculeata* (Gorham, 1887), comb. nov. **A–D** lectotype of *T. aculeata*: **A** dorsal habitus **B** ventral habitus **C** lateral view **D** labels **E–H** paralectotype of *T. aculeata*: **E** dorsal habitus **F** ventral habitus **G** lateral view **H** labels **I–L** second paralectotype of *T. aculeata*: **I** dorsal habitus **J** ventral habitus **K** lateral view **L** labels. Scale bars: 2mm.

Antenna with 11 antennomeres and club composed of five fringed antennomeres. Antennomere III almost as long as antennomere IV, antennomere V subequal to antennomere VI, both of them shorter than each of antennomeres III or IV, antennomere VII subtriangular but slightly dilated, antennomeres VIII–XI dilated, apex of antennomere XI rounded. Head with fine punctation, nearly smooth. Clypeus broader than long. Eyes large, finely faceted. Mandibles robust and with outer side nearly straight.

**Figure 2.**
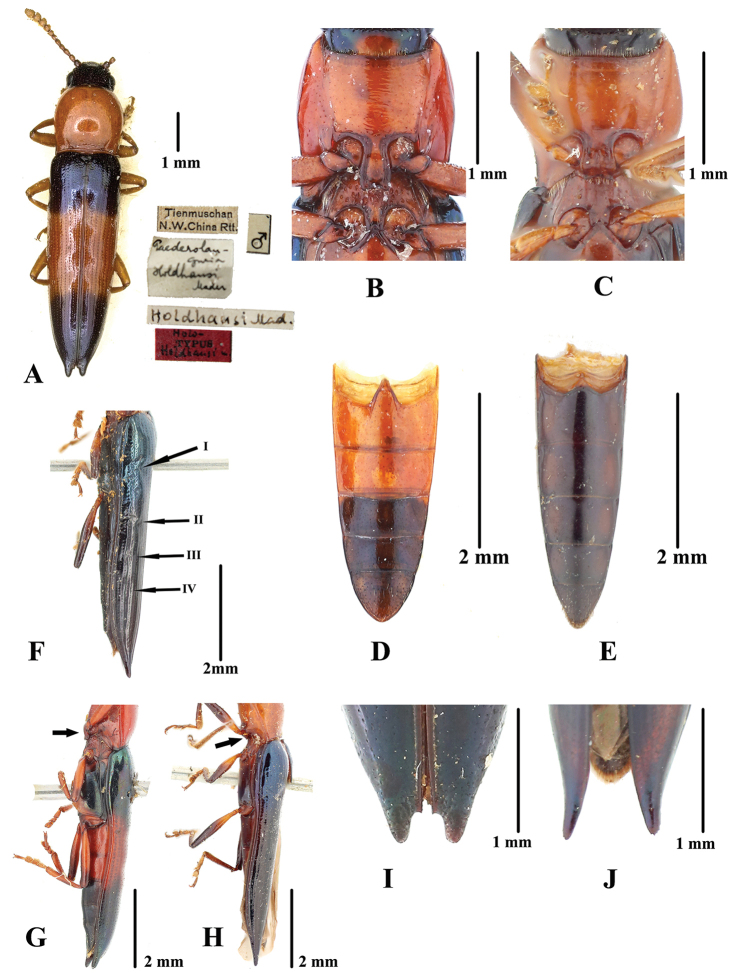
Comparison between *Paederolanguria holdhausi* Mader, 1939 and *Tomolanguria aculeata* (Gorham, 1887), comb. nov. **A** Holotype of *Paederolanguria holdhausi* Mader, 1939 **B***P. holdhausi*, prosternum and mesoventrite with punctures, non-type **C***T. aculeata*, prosternum and mesoventrite without punctures, paralectotype **D***P. holdhausi*, abdomen, non-type **E***T. aculeata* comb. nov., abdomen, paralectotype **F** lateral view of *T. aculeata*, black arrows show the depressions on the elytron, paralectotype **G** later view of body of *P. holdhausi*, black arrow shows the prosternal process nearly straight, non-type **H** same of *T. aculeata*, black arrow shows the prosternal process downwards, paralectotype **I***P. holdhausi*, apex of the elytra, non-type **J***T. aculeata* comb. nov., apex of elytra, paralectotype. Scale bars: 1 mm (**A–C, I, J**); 2 mm (**D–H**).

Pronotum slightly convex, distinctly longer than broad, with sides rounded, constricted at the base. Pronotum finely punctured, without basal foveae. Anterior angle rounded, posterior angle acute but not produced. Lateral and basal margins beaded. Prohypomera smooth, without punctation or folds. Prosternal process long, with apex strongly bent downwards. Procoxal cavities open. Mesoventrite without punctation, nearly smooth (Fig. 2C).

Scutellar shield short and cordiform, apex not acute. Elytra regularly striate-punctate, vanishing before apex. Elytral epipleura distinct, narrow. Elytra at humeri slightly broader than base of pronotum. Sides of elytra parallel, narrowed posteriorly. Each elytron with four shallow transverse depressions (Fig. 2F). Elytral apex distinctly produced and acute, laterally curved, with four or five coarse denticles (Fig. 2J). Abdomen finely punctured, postmetacoxal lines absent (Fig. 2E).

##### Material examined.

***Lectotype*** (BMNH), female, here designated, labeled: “Type” [circular label with red margin]// “Type. /Sp. figured.” // “S. And. Tuxtla” // “Mexico./ Salle Coll.”// “2336” [green label]// “*Languria/ aculeata* Chev./ apud Sallé” [handwriting]// “B. C. A., Col., VII./ *Languria/ aculeata*, Gorh.”(Fig. 1D). ***Paralectotypes*** (BMNH), two females, with the same labels (Fig. 1H, L).

##### Distribution.

Mexico, San Andrés Tuxtla.

## Discussion

**Figure 3.**
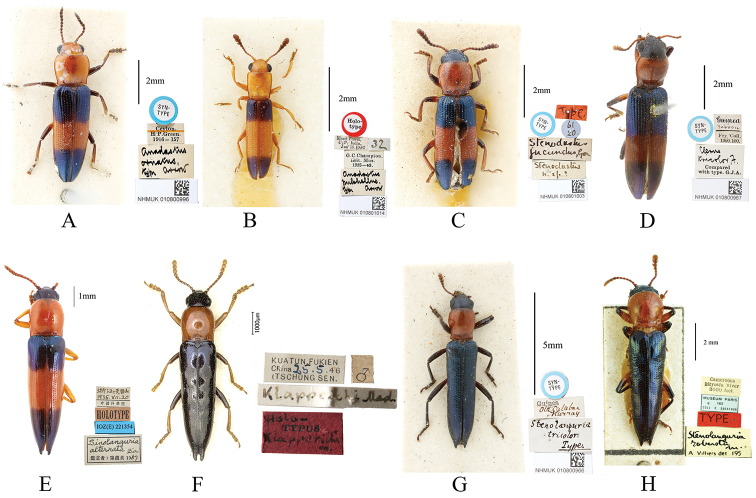
Body of species from different genera of Languriinae that may imitate genus *Paederus*. **A***Anadastus ornatus* Arrow, 1925, syntype **B***A. pulchellus* Arrow,1925, holotype **C***A. jucundus* (Gorham, 1903), syntype, synonym of *A. bifasciatus* (Motschulsky, 1860) **D***Clerolanguria tricolor* (Fabricius, 1787), syntype **E***Paederolanguria alternata* (Zia, 1959), holotype **F***P. klapperichi* Mader, 1955, holotype **G***Stenolanguria tricolor* Fowler, 1885, syntype **H***S. robusta* Villiers, 1958, holotype. Scale bar: 2 mm (**A–D, H**); 1 mm (**E, F**); 5 mm (**G**).

The potential mimicry between Languriinae and Staphylinidae has been recorded (Mader 1939; Reid and Noerdjito 1994). When Mader (1939) erected the genus *Paederolanguria*, which was named after the genus *Paederus* Fabricius, 1775, he designated *P. holdhausi* (Fig. 2A) as the type species and mentioned that it displayed a color pattern similar to that in species of *Paederus*. (Staphylinidae). Reid and Noerdjito (1994) also reported a similarity between an undetermined species of Languriinae and *Paederus* from Java. The specimens examined in this study and an additional search of the Internet shows that such a mimic phenomenon is not rare in Languriinae and that it demonstrates two different forms. Some languriines show only the characteristic color pattern of *Paederus*, with red and alternative blue or dark stripes, including several species from the genus *Anadastus* Gorham, 1887 (Fig. 3A–C): *Clerolanguria tricolor* (Fabricius, 1787) (Fig. 3D), and *Languria trifasciata* Say, 1823. Some others not only have the similar color pattern but also somewhat modified structure, such as their bodies are more slender, with antennae not very dilated, pronotum rounded, elytra with weak transverse depressions, and elytral apex more or less produced or acute, which can be observed in species from the genera *Paederolanguria*, (Fig. 3E, F), *Stenolanguria* (Fig. 3G, H), and *Tomolanguria* (Fig. 1A, E, I).

**Figure 4.**
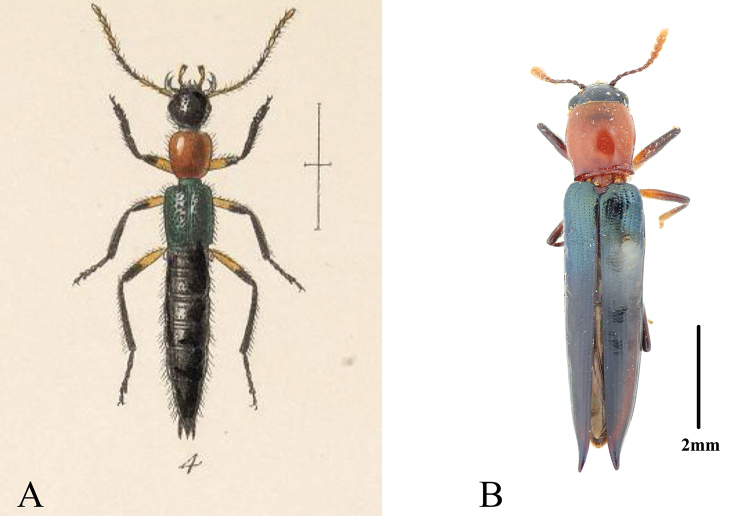
Comparison between *Paederus signaticornis* Sharp, 1886 and *Tomolanguria aculeata* (Gorham, 1887), com. nov. **A** habitus of *P. signaticornis* (from Sharp 1886: pl. XVI, fig. 4) **B** habitus of *T. aculeata* comb. nov. Scale bars: 2 mm.

After discussion with Dr José Luis Navarrete-Heredia, a specialist of Staphylinidae, we suggest that *Paederus signaticornis* Sharp, 1886 should be a suitable model for *Tomolanguria aculeata* (Fig. 4A, B). Both *P. signaticornis* and *T. aculeata* share the same distribution in Mexico, and the elytral color pattern of *T. aculeata* matches with that of *P. signaticornis*. For example, the basal third of the elytra of *T. aculeata* with a green metallic luster mimics the elytral color of *P. signaticornis*, the remaining part of the nearly black elytra mimics the color of the staphylinid abdomen. Besides, the color pattern and depressions on the elytra probably mimic the segmentation of the abdomen, and the produced apex of elytra may mimic tergite IX of *P. signaticornis*.

The genus *Paederus* is famous for their toxic, paederus dermatitis (Frank and Kanamitsu 1987). Kellner and Dettner (1996) suggested that the toxin is the basis for prey rejection. Their classic color pattern may be an example of aposematism, a warning signal to potential predators. Languriines may imitate the warning signals, even the appearances of *Paederus*, as an anti-predator strategy. In summary, we suppose that there may be a potential mimic relationship between this new genus *Tomolanguria* and genus *Paederus*.

## Supplementary Material

XML Treatment for Tomolanguria

XML Treatment for Tomolanguria aculeata
